# Life-Threatening Hypocalcemia following Subtotal Parathyroidectomy in a Patient with Renal Failure and Previous Roux-en-Y Gastric Bypass Surgery

**DOI:** 10.1155/2011/370583

**Published:** 2011-10-29

**Authors:** Betsy Palal, Marvin Sinsakul, Sirimon Reutrakul

**Affiliations:** ^1^Section of Endcrinology, Department of Medicine, Rush University Medical Center, Chicago, IL 60612, USA; ^2^Section of Nephrology, Department of Medicine, Rush University Medical Center, Chicago, IL 60612, USA

## Abstract

*Background.* Roux-en-Y gastric bypass (RYGB) can result in calcium and vitamin D deficiency. Parathyroid surgery carries the risk of immediate and long-term hypocalcemia. *Methods and Results.* We describe a 54-year-old woman with history of end-stage renal disease and gastric bypass surgery who developed calciphylaxis requiring a 3.5-gland parathyroidectomy. Seven weeks later, she presented with weakness, perioral numbness, leg cramps, a positive Chvostek's sign, hypotension, prolonged QT-interval, and serum calcium of 5.4 mg/dL. Oral and intravenous calcium, calcitriol, and high calcium bath hemodialysis were given. She required 18 days of intravenous calcium and an outpatient maintenance regimen of calcitriol 6 mcg/day, calcium carbonate 8 grams/day, calcium citrate 1.2 grams/day, and ergocalciferol 50,000 IU/week. *Conclusion.* The patient's life-threatening prolonged hypocalcemia and large requirements of calcium and calcitriol were due to a combination of malabsorption, hypoparathyroidism, and renal failure. Special considerations should be given to bariatric surgery patients undergoing neck exploration.

## 1. Introduction

The prevalence of obesity in the United States has drastically increased over the last several decades. The percentage of adults who are obese (BMI ≥ 30) increased from 15.3% in 1995 to 23.9% in 2005, with 4.8% considered to be morbidly obese (BMI ≥ 40) [1]. The use of bariatric surgery, first introduced in 1950, has increased dramatically as a way to treat this problem, from 16,000 procedures in 1997 to more than 100,000 in 2003 [2]. The surgery resulted in long-term weight loss and reduction in total mortality especially from diabetes, cancer, and heart disease [3, 4].

Roux-en-Y gastric bypass (RYBG) and biliopancreatic diversion (BPD) are the two commonly performed operations. Because of the malabsorptive nature of the procedures, they can result in protein malnutrition as well as vitamin and mineral deficiency (calcium and vitamin D, iron, folate, thiamine, vitamin B 12, and vitamin A) [5]. Specifically, these procedures bypass the duodenum and jejunum, which are the preferential sites for calcium absorption. Overall, 10–25% of patients develop calcium deficiency by 2 yr and 25–48% by 4 yr; 17–52% of patients develop a vitamin D deficiency by 2 yr and 50–63% by 4 yr [5–11]. As a result, the body upregulates parathyroid hormone (PTH; secondary hyperparathyroidism), causing increased production of vitamin D and calcium resorption from the bone and helping to maintain calcium level. These patients, therefore, need regular monitoring and supplementations of calcium and vitamin D. 

Secondary hyperparathyroidism commonly develops in chronic renal failure. Elevated PTH is a result of hyperphosphatemia, due to reduction in phosphate excretion, and hypocalcemia, due to impaired conversion of 25-hydroxy vitamin D to the active form 1,25-dihydroxy vitamin D and decreased intestinal absorption of calcium. This is usually medically managed by phosphate binders, vitamin D analogues, and calcimimetics such as cinacalcet to reduce serum phosphate and PTH levels. However, about 1-2% of patients require parathyroidectomy each year due to failure of medical management or the development of calciphylaxis [12]. This occurs when the levels of calcium and phosphate in the blood exceed their solubility level, leading to calcium-phosphate deposits in small- and medium-sized arteries causing ischemia and necrosis of skin and soft tissue. Calciphylaxis is associated with high mortality of 60–80% [13, 14] due to infection and ulcerations.

Parathyroid surgery carries the risk of immediate and long-term hypocalcemia. This can become more complicated when parathyroidectomy is performed in patients with multiple comorbidities that affect calcium and vitamin D metabolism. Here, we describe a patient who had undergone an RYGB, and later developed end-stage renal disease (ESRD). She underwent a 3.5-gland parathyroidectomy for a calciphylaxis of the breast and developed life-threatening prolonged hypocalcemia.

## 2. Materials and Methods

We reviewed the patient's clinical and laboratory findings, and clinical course from her medical record. Literature reviewed and possible mechanisms are provided.

## 3. Results

A 54-year-old female with significant past medical history of ESRD on hemodialysis underwent a 3.5-gland parathyroidectomy due to severe calciphylaxis of her breast which required multiple debridements. She had a history of RYGB five years prior due to morbid obesity (BMI 71 kg/m^2^). Prior to the parathyroidectomy, her calcium levels were 8.3–9.8 mg/dL (reference range 8.7–10.7), phosphorus 4.7 mg/dL (reference range 3.9–6.6), magnesium 1.7–2.4 mg/dL (reference range 1.6–2.7), PTH 1971 pg/mL (reference range 22–60), and serum creatinine 5.6 mg/dL (reference range 0.5–1.1). Vitamin D status was not known. She had been treated with sevelamer and cinacalcet in the attempt to control secondary hyperparathyroidism.

During surgery, after all four parathyroid glands were identified, intraoperative PTH (ioPTH) was 2466 pg/mL. A 3.5-gland parathyroidectomy was performed. Ten minutes later, an ioPTH was 119 pg/mL. This was deemed to be satisfactory. Cryopreservation of parathyroid tissue was not performed. On postoperative day 1, calcium was 7.0 mg/dL and ionized calcium was 0.7 mmol/L (reference range 0.95–1.32) and PTH was 49 pg/mL. She was discharged on calcitriol 0.5 mcg/day and calcium carbonate 9 grams/day. Pathology revealed hyperplastic parathyroid glands.

**Figure 1:**
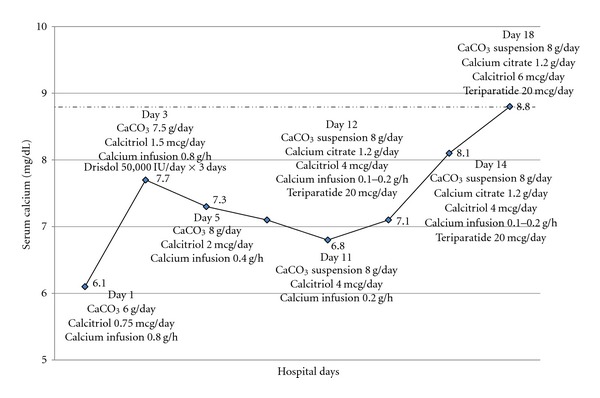
Calcium levels and treatment during the hospital course.

Seven weeks later she presented to the emergency department with complaints of generalized weakness, perioral numbness, and leg cramps for several days. Her physical examination was remarkable for BP 140/70 mmHg, a positive Chvostek's sign and a healing right breast ulcer. An EKG revealed a prolonged QT-interval. Her calcium level was 5.4 mg/dL with a PTH level of 87 pg/mL, phosphorus 3.1 mg/dL (reference range 2.5–4.6), and magnesium 2.1 mg/dL (reference range 1.6–2.7). She was treated with oral calcium carbonate 6 grams/day, in divided doses, calcitriol 0.75 mcg/day, and a continuous calcium gluconate infusion. Overnight, the patient developed hypotension (BP 60/40 mmHg) requiring a transfer to the intensive care unit and an initiation of vasopressor. The detail of her hospital course is shown in Figure 1.

 After 48 hours, calcium increased to 8.3 mg/dL, with an improvement of blood pressure and the vasopressor was stopped. Hemodialysis with a high calcium bath was performed. When attempting to hold the calcium infusion, her calcium dramatically dropped to 5.0 mg/dL, along with intermittent episodes of hypotension, requiring the infusion to be restarted. Calcitriol was increase to 1.5 mcg/day on hospital day 3. On hospital day 4, her oral calcium carbonate was increased to 7.5 grams/day and ergocalciferol 50,000 IU weekly was added for a 25-hydroxy vitamin D level of 16 ng/mL (reference range 20–100). 

 On hospital day 11, calcium and calcitriol supplementations were changed to suspension and solution forms due to concern of the patients' inability to absorb the tablet form. Multiple attempts to wean off the calcium infusion were unsuccessful. On hospital day 12, teriparatide 20 mcg/day was started. Eventually, her calcium level and blood pressure became more stabilized and she was transferred from the ICU to the medical floor after two weeks. She required a total of 18 days of continuous calcium infusion. The patient was discharged on calcium carbonate suspension 8 grams/day, calcium citrate suspension 1.2 grams/day, calcitriol solution 6 mcg/day, ergocalciferol 50,000 IU weekly, and teriparatide 20 mcg/day. She continued on a high calcium bath dialysis. At her follow-up visit 4 weeks later her calcium was 9.7 mg/dL, and teriparatide was stopped. Her calcium levels remained stable between 8.4 and 9.0 mg/dL on the same regimen nine months later. Phosphorus levels were 3.2–5.0 mg/dL, magnesium 2.2–3.5 mg/dL, PTH 79–115 pg/mL, and 25-hydroxy vitamin D 37–50 ng/mL during the same period.

## 4. Discussion

This case demonstrates the risk of severe hypocalcemia in bariatric patients who develop hypoparathyroidism. To our knowledge, this is the first reported case of life-threatening prolonged hypocalcemia after parathyroidectomy in the setting of previous RYGB and ESRD. This was due to a combination of malabsorption, hypoparathyroidism, ESRD, and hungry bone syndrome. While any of these four etiologies can cause hypocalcemia, our case is the first to describe these combinations and highlights the importance of different body systems in regulating calcium levels.

Hypocalcemia and vitamin D deficiency after a malabsorptive bariatric surgery are due to multiple reasons. The duodenum and jejunum, the preferential sites for calcium absorption, are bypassed. In the setting of low calcium intake, the duodenum can absorb 80–100% of calcium by a vitamin D-dependent transcellular active transport. When this is bypassed, the calcium absorption takes place through the less efficient paracellular mechanism [15]. Partial gastrectomy reduces gastric acidity, resulting in an impaired absorption of calcium salts. Malabsorption of fat-soluble vitamins due to poor mixing of bile salts decreases the amount of vitamin D available and contributes further to the decreased calcium absorption [11]. In addition, gastric bypass patients consume only about 50% of the recommended daily requirements of vitamin D unless taking regular supplement, and many cannot tolerate calcium-rich diets [16, 17]. Therefore, these patients should have calcium and vitamin D, as well as phosphorus, PTH, and alkaline phosphatase levels done preoperatively and every 6 months thereafter [5]. Yearly bone mineral density measurement is recommended until stable. Daily calcium 1.2–2 grams/day, preferably calcium citrate with vitamin D should be given [5]. Vitamin D supplementation can be provided with ergocalciferol, 50,000 IU one to three times weekly. Therapy should be adjusted based on serum markers and measures of bone density.

Renal failure-related secondary hyperparathyroidism occurs in as many as 90% of patients by the time hemodialysis is initiated [18], and is usually medically managed. When medical management fails (persistently elevated PTH > 800 pg/mL, hyperphosphatemia with calcium × phosphorus >70, hypercalciuria, hypercalcemia, pathologic fractures, ectopic soft tissue or vascular calcifications, or calciphylaxis), parathyroidectomy should be considered [19]. The recent surgery rate in 2007, however, has decreased from previously reported in 2002, likely due to improved medical treatment and guideline publication [20]. Preoperative localization studies are not always routinely performed, with the exception of reoperation, due to the low sensitivity of ultrasound and ^99m^Tc-sestamibi scintigraphy in patients with multiple-gland disease as seen in renal failure. In addition, the ability of imaging techniques to identify ectopic parathyroid is limited in this circumstance. Therefore, bilateral neck exploration is the standard of care for these patients [19]. Three different surgical approaches have been described, including subtotal parathyroidectomy (removal of 3.5 glands leaving a remnant *in situ*), total parathyroidectomy with autotransplantation, and total parathyroidectomy without autotransplantation. The cure rate was higher in patients after total parathyroidectomy with autotransplantation than subtotal parathyroidectomy, 100% versus 90% [21, 22]. More recently, total parathyroidectomy without autotransplantation has been proposed and shown to be associated with a lower rate of recurrence than those with autotransplantation [23]. When performed, autotransplantation in the forearm is preferred for the convenience of reoperation in case of the disease recurrence [24, 25]. Routine cryopreservation is advocated by some although the overall utility rate of cryopreserved parathyroid tissue is estimated at 1.6%, with significantly decreased tissue viability after 24 months [19, 26, 27]. Data on the utility of ioPTH in these patients is limited and difficult to evaluate [28]. Different criteria have been shown to predict postoperative cure in different studies, such as a 50% decrease from baseline at 10 minutes [29], >90% decrease at 15 minutes [30], and PTH < 45 pg/mL at 30 minutes [31]. 

While many patients with ESRD do well after total or subtotal parathyroidectomy, with 15–30% experiencing transient hypocalcemia and rarely long-term hypocalcemia [19, 32], this was not the case in our patient. Due to calcium and vitamin D malabsorption associated with RYGB, her PTH played a pivotal role in maintaining her calcium level preoperatively. Subsequent parathyroidectomy resulted in severe hypocalcemia requiring large doses of calcium and vitamin D supplement. The inability to convert 25-hydroxy vitamin D to 1,25-dihydroxy vitamin D due to renal failure made it more difficult to normalize her calcium levels. While her initial large calcium and calcitriol requirement could be due to a hungry bone syndrome, this was unchanged during a follow-up period indicating an ongoing malabsorption. Our literature review found few reported cases of severe hypocalcemia, requiring prolonged intravenous calcium infusion and large doses of oral calcium and vitamin D supplement, in RGBY or BPD patients following thyroid surgery complicated by hypoparathyroidism, although none had ESRD [33–36]. Our patient received a short trial of teriparatide injection after difficulty weaning the calcium infusion, given previous reports in patients with primary hypoparathyroidism [37, 38]. It was difficult to determine the effectiveness of this treatment as there were simultaneous adjustments in calcium and vitamin D doses. Delayed autotansplantation might have been considered had the parathyroid tissue been cryopreserved. During outpatient followup, the patient continued to have serum calcium, phosphate, magnesium, and vitamin D levels monitored regularly as recommended in hypoparathyroidism patients [39]. Routine monitoring of urinary calcium and prevention of hypercalciuria is usually recommended, except our patient was anuric.

Thyroid and parathyroid surgery, as well as bariatric surgery are common procedures. Physicians should be aware of an increased risk of severe hypocalcemia in gastric bypass patients who subsequently develop hypoparathyroidism and the requirement of larger than usual doses of calcium and calcitriol supplementations. Precautions should be taken when these patients undergo neck exploration. Optimization of calcium and vitamin D repletion prior to surgery, as well as aggressive supplement immediately postoperatively, may be helpful.
